# Liquid Crystalline Network Composites Reinforced by Silica Nanoparticles

**DOI:** 10.3390/ma7075356

**Published:** 2014-07-22

**Authors:** Zhen Li, Yang Yang, Benye Qin, Xiaoyong Zhang, Lei Tao, Yen Wei, Yan Ji

**Affiliations:** Department of Chemistry, Tsinghua University, Beijing 100084, China; E-Mails: chemlizhen@gmail.com (Z.L.); menghuan0109@126.com (Y.Y.); qinbenye@gmail.com (B.Q.); xiaoyongzhang1980@gmail.com (X.Z.); leitao@mail.tsinghua.edu.cn (L.T.)

**Keywords:** liquid crystalline network, composite, silica nanoparticle

## Abstract

Liquid crystalline networks (LCNs) are a class of polymers, which are able to produce mechanical actuation in response to external stimuli. Recent creation of LCNs with exchangeable links (xLCNs) makes LCNs easy moldable. As the xLCNs need to be shaped at a high temperature, it is important to enhance their thermal and mechanical properties. In this paper, a series of xLCNs/SiO_2_ composites containing 1%–7% SiO_2_ nanoparitcles (SNP) were prepared and their thermal and mechanical properties were examined. The results show that xLCNs/SNP composites have lower liquid crystalline-isotropic phase transition temperature and higher decomposition temperature than pure LCN. The tensile strength and the elongation at break of xLCNs at high temperatures were also enhanced due to the addition of SNPs.

## 1. Introduction

Liquid crystalline networks (LCNs) are a type of smart materials which have the ability to convert external stimuli into mechanical response [1,2,3]. Due to such a capability and other remarkable physical characteristics, such as softness, lightweight, and so on [4], LCNs have been proposed as a promising candidate for many applications, such as muscle mimics [5,6,7], motors [8], contact lenses [9], microgrippers [10], microvalves [11], haptic devices [12], and tunable lasing media [13]. Usually the macroscopic orientation of liquid crystal order is hard to achieve, which has greatly limited their application in practice. Recently, the permanent network crosslinks were replaced by exchangeable links, resulting in the xLCNs [14]. Not only can the xLCNs be molded and remolded into various shapes, but also they can be aligned conveniently. As the xLCNs materials need to be processed at high temperatures, it is desirable to enhance their thermal and mechanical properties.

The incorporation of nanoparticles into LCNs is a commonly way to improve the LCN’s performance. For examples, as carbon nanotubes (CNTs) are capable of converting light into heat efficiently, the LCNs became sensitive to light after the addition of CNTs [15,16]. The silica coated nanoparticles can also act as light absorber [17]. Schmidt’s group reported the incorporation of superparamagnetic Fe_3_O_4_ nanoparticles into LCN. Due to hysteresis, the materials exhibited actuation upon the application of an alternating magnetic field [18]. Anisotropic magnetic nanoparticles were also incorporated into the LCN [19]. Other nanoparticles, such as PbTiO_3_ [20], Molybdenumoxide [21], were also reported. There are investigations on the mechanical and thermal properties of the LCN and nano particles (NPs) composites [22]. In general, the presence of nanoparticles lowers liquid crystalline-isotropic phase transition temperature (Ti) and glass transition temperature (Tg) slightly, while increasing the stiffness of the materials. However, the corporation of SiO_2_ nanoparticles into LCN, especially “xLCN” with the exchangeable links, has not been investigated.

In many cases, SiO_2_ nanoparticles were incorporated to reinforce polymeric materials. Chengfang Ou [23] reported three series of epoxy/SiO_2_ composites. Compared to pure epoxy, these composites had higher Tg and decomposition temperature (Td). Alzina* et al.* modified the SiO_2_ with epoxide functional groups and examined the thermal properties of the composite. In this system, the Tg and Td decreased in the presence of SiO_2_ [24].

In this paper, a series of xLCNs/SiO_2_ composites (xLCN/SNPs), which contained 1–7 wt% SiO_2_ nanoparticles (SNP) were obtained. The thermal and mechanical tests show that xLCNs/SNP composites have lower Ti and higher Td than the pure xLCN. There is also an improvement on the tensile strength and the elongation at break due to the addition of SNP.

## 2. Results and Discussion

### 2.1. The Glass and Isotropic Transition Temperatures

**Figure 1:**
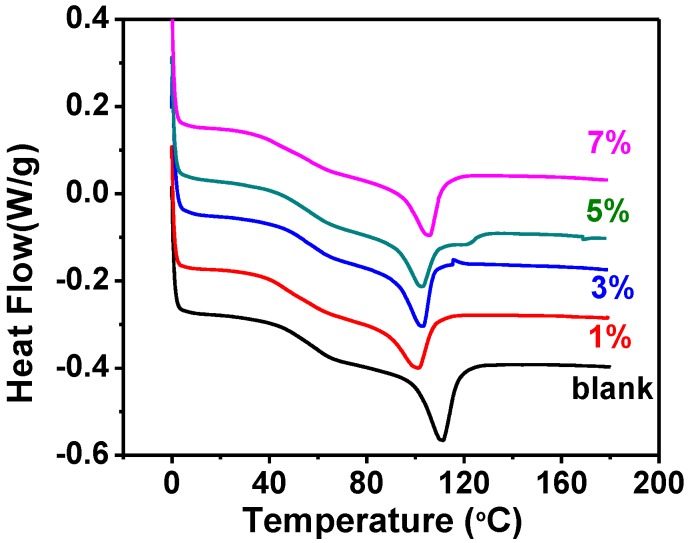
DSC thermograms of xLCNs/SiO_2_ composites (xLCN/SNP) and blank xLCN.

DSC was utilized to determine the Tg and Ti of these composites. Figure 1 shows the DSC thermograms of the xLCN/SNP composites and the blank xLCN. Two thermal transitions appeared in each sample: a Tg and a Ti. An apparent difference of the Ti location can be seen from the curves. To obtain accurate value of Ti, the DSC measurements at different heating/cooling rate were taken and the Ti value was calculated by extrapolating the values to zero rate (Figure S1). As shown in Table 1, every Ti of the xLCN/SNP samples was lower than that of the blank xLCN. This is probably because nanoparticles act like impurities, disturbing the liquid crystal orientation [17,25,26]. Meanwhile, as the nanoparticles content increased, the Ti increased too. A possible explanation is that the SNPs also restrict the motion of the molecules. Similar to Ti, such phenomenon was observed for Tg.

**Table 1:** Thermal data of xLCNs/SiO_2_ composites (xLCN/SNP) and blank xLCN sample.

Sample	Tg (°C)	Ti (°C)	Td (°C)	Ti enthalpy (J/g)	Weight at 800 °C (%)
blank	60.2	114.3	329.5	7.93	0
SNP 1%	46.1	104.1	360.1	6.05	1.49
SNP 3%	54.4	105.2	340.4	6.64	3.12
SNP 5%	57.4	108.2	357.3	7.20	3.87
SNP 7%	56.8	107.9	356.2	7.06	5.92

### 2.2. Thermal Stability

**Figure 2:**
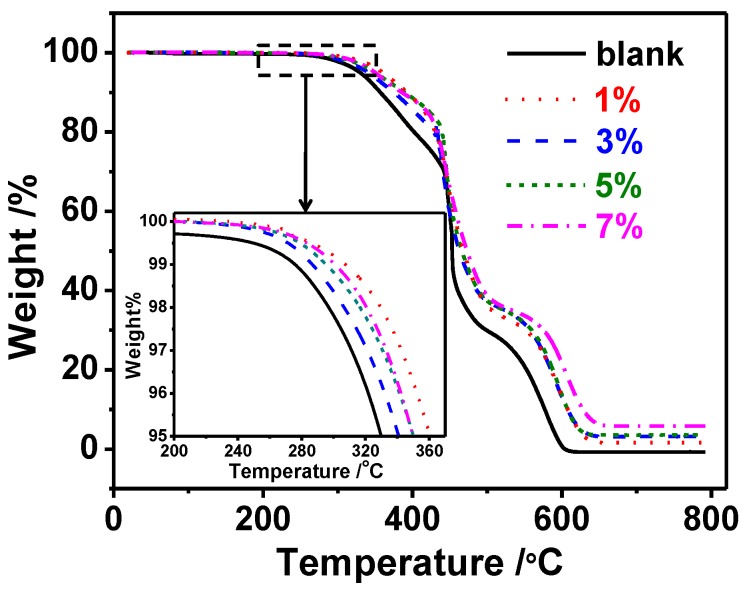
TGA curves of xLCNs/SiO_2_ composites (xLCN/SNP) and blank xLCN sample.

Thermogravimetric analyses of thermoset nanocomposites are presented in Figure 2. The degradation of pure xLCN started at about 275 °C. When silica nanoparticles were added, the material degraded at higher temperatures. As can be seen from Figure 2, the Td of xLCN with 1% SNP was about 30 °C higher than that of the blank one. All the samples doped with silica nanoparticles were more stable than the blank sample. The weight percent at 800 °C of all samples were listed in Table 2, these values largely depended on the SNP content, and as the SNP increased, the weigh value at 800 °C increased too. Although there was not much relevance observed between Td and SNP contents, it still could be concluded that the overall thermal stability was improved by the addition of SiO_2_ nanoparticles.

**Table 2:** Young’s modulus of xLCNs/SiO_2_ composites (xLCN/SNP) and blank xLCN sample at different temperatures.

Temperature (°C)	Blank	SNP 1%	SNP 3%	SNP 5%	SNP 7%
30	3.636	2.674	3.063	3.208	2.494
90	0.0454	0.0608	0.0557	0.0501	0.0447
160	0.0151	0.0182	0.0143	0.0199	0.0178

### 2.3. Dynamic Mechanical Properties of the Composites

**Figure 3:**
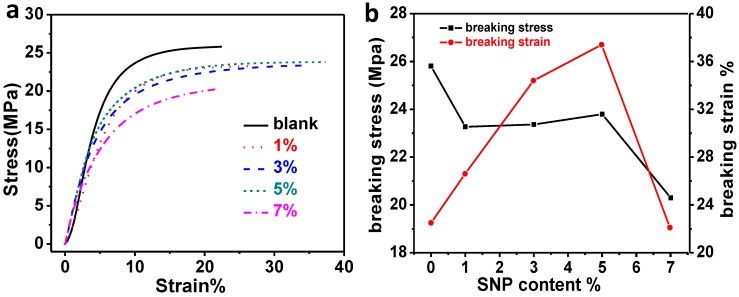
Stress-Strain curves and breaking stress/strain plot of xLCNs/SiO_2_ composites (xLCN/SNP) and blank xLCN samples at 30 °C.

The mechanical properties of the composites were investigated by a tensile test with the force increased by a rate of 0.1 N/min from 0.1 N to 18 N at different temperatures. Figure 3a shows the stress-strain curves of xLCN/SNP and blank xLCN samples at 30 °C. At this temperature, pure LCN always had higher Young’s modulus. As shown in the picture, the blank sample had smaller strain than the xLCN/SNP composites under the same stress. However, as shown in Figure 3b, compared to the xLCN/SNP composites, the blank sample broke easily at lower strain. When silica nanoparticles were added, the modulus of the material reduced a bit but a bigger elongation at break was achieved for most samples.

**Figure 4:**
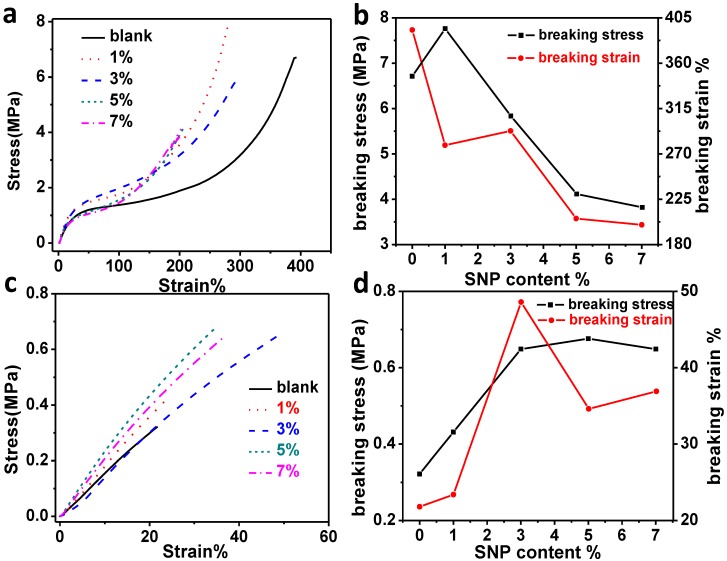
Stress-Strain curves and breaking stress/strain plot of xLCNs/SiO_2_ composites (xLCN/SNP) and blank xLCN sample at (**a**,**b**) 90 °C and (**c**,**d**) 160 °C.

Since xLCNs need to be reshaped at a temperature above Tg to get a temporary shape and above 160 °C for a permanent shape, it is important to evaluate the mechanical properties of those composites at those high temperatures. As can be seen in Figure 4a, all the xLCN/SNP composites can stand larger stress than the pure xLCNs sample at the same strain, but the sample with excessive nanoparticles (>5%) would break more easily. As presented in Figure 4c, the mechanical properties of xLCN/SNP composites were all better than the blank sample at 160 °C. At this temperature, the sample with SNP content can stand bigger stress and larger strain before break, which can be seen in Figure 4d. As shown in Table 2, compared to the blank sample, most of the xLCN/SNP composites have bigger Young’s modulus. One possible explanation for this phenomenon is that as the temperature rises over Tg or Ti, the interaction between polymer chains get weaker, and the interaction between SNP and polymer become to play a more important role [27].

### 2.4. Morphology Investigation of Fractured Surface

**Figure 5:**
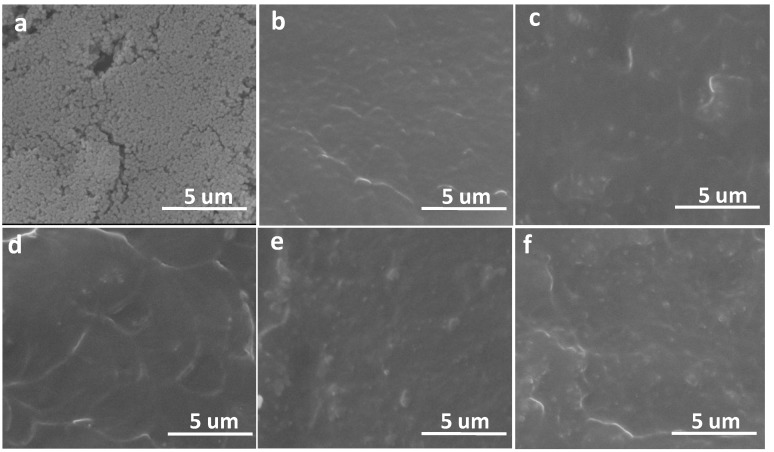
SEM micrographs of (**a**) silica nanoparticles and the fracture surfaces of (**b**) blank samples and the xLCNs/SiO_2_ composites (xLCN/SNP) composites with (**c**) 1%; (**d**) 3%; (**e**) 5%; (**f**) 7% content.

SEM micrographs were recorded to investigate the agglomeration of the SiO_2_ nanoparticles in polymer matrix, which has an important influence on the mechanical properties [28]. As presented in Figure 5a, the silica nanoparticles are narrowly dispersed with a polydispersity of 0.06 (Figure S2). A SEM image of the nanoparticles at a magnification of 30,000 was presented in Figure S3. Figure 5b–f shows SEM micrographs of the fracture surfaces of samples which were prepared in liquid nitrogen. The cross section of blank sample seems clean and homogeneous. As shown in Figure 5c,d, for the sample with lower content of SNP, the cross section was similar like the blank sample and agglomeration can be hardly observed by the small white dots. As the content of SNP increased, the fracture surface gets rough and small cluster can be observed, indicating the agglomeration. However, the clusters are far smaller than 1 μm and many isolated SiO_2_ nanoparticle dots can be observed, which indicates a good dispersion.

### 2.5. Thermal Actuation and Shape Memory

**Figure 6:**
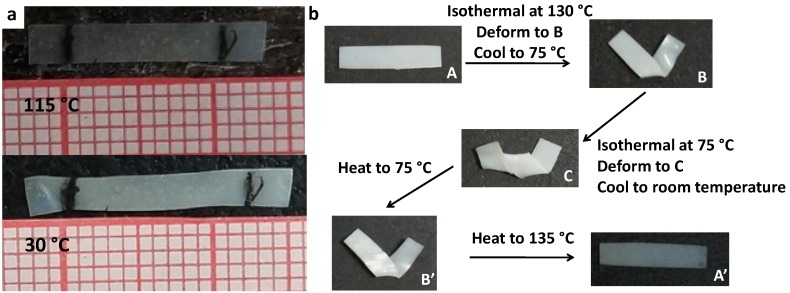
(**a**) The change of natural length of a free-standing monodomain xLCNs/SiO_2_ composites (xLCN/SNP) (5 wt%) between the liquid-crystal phase (30 °C) and the isotropic phase (115 °C); (**b**) Triple shape memory of a polydomain xLCNs/SiO_2_ composites (xLCN/SNP) (5 wt%) sample.

Like the blank sample, all the monodomain SiO_2_ dispersed xLCNs can show the similar thermal reversible actuation and the polydomain samples exhibit the irreversible triple shape memory effect. For example, a free-standing monodomain xLCN/SNP (5%) sample was prepared by the method reported before [14]. Figure 6a shows the change of natural length of the monodomain xLCN/SNP (5%) film at different temperatures. At the temperature (115 °C) above Ti, the sample strip was in isotropic phase. When it cooled down to room temperature (30 °C), the sample elongated to an uniaxially aligned strip. The triple shape-memory of the polydomain xLCN/SNP (5%) was demonstrated in Figure 6b. The sample with a flat shape (A) was deformed into a V-shape (B) at 130 °C. After cooling down to 75 °C, this shape was fixed temporarily. Then the sample was further deformed at 75 °C into a new shape (C), which was then fixed by cooling below Tg. During the heating process, the sample recovered the shape B (above Tg) and then the original shape A (above Ti) successively. As shown in this experiment, xLCN still have the triple shape memory effect and the corresponding monodomain maintains the properties with the absence of SNPs.

## 3. Experimental Section

### 3.1. Preparation of SiO_2_ Nanoparticles

Silica nanoparticles (SNP) were synthesized using the classic Stober method [29]. Catalyzed by ammonia, tetraethyl orthosilicate (TEOS) were hydrolyzed in ethanol. An amount of 1.9 g aqueous ammonia solution containing 28% NH_4_OH was diluted in technical ethanol (96%) to 20 mL. An amount of 8.33 g TEOS was dissolved in technical ethanol to 20 mL and was slowly added to the NH_4_OH solution. Then the mixture was stirred overnight at room temperature. The product was separated by centrifugation at 8000 rpm and dried in vacuum at 60 °C. Dynamic light scattering (DLS) showed the nanoparticles have a size of 250 nm.

### 3.2. Preparation of xLCNs/SNP Film

**Scheme 1:**
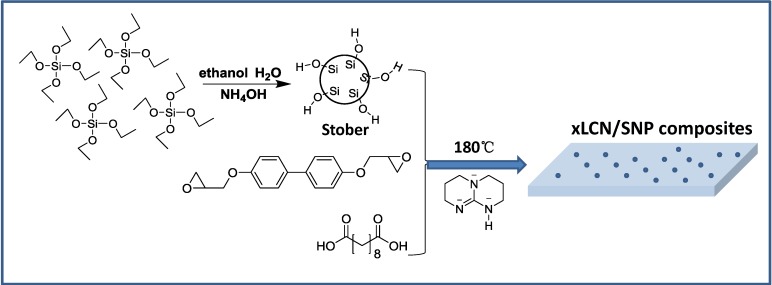
Sketch of the chemical process to obtain xLCNs/SiO_2_ composites (xLCN/SNP).

The process for the synthesis of the LCN/SNP composites is presented in Scheme 1. An amount of 0.298 g 4,4'-dihydroxybiphenyl, 0.202 g sebacic acid, and various SNP content (1%–7%) were dispersed in chloroform and sonicated for 5 min. Then the mixture was evaporated and melt at 160 °C before a triazabicyclodecene catalyst (5 mol% to the COOH groups) was introduced and stirred manually until homogeneous. Then the mixture was cured at 180 °C for 4 h between two glass slides covered with polytetrafluoroethylene tape. The monodomain preparation procedure is exactly the same as that used in former paper [14], which has given all the detailed information.

### 3.3. Characterization

DSC measurements were performed on TA 2000 analyzer (TA Instruments, New Castle, PA, USA). Appropriate amount of samples were heated from 20 °C to 180 °C at a temperature scanning rate of 10 °C/min under dry nitrogen atmosphere. The DSC measurements at different heating/cooling rate of 5 °C/min, 10 °C/min, 15 °C/min, 20 °C/min, 25 °C/min were taken and the Ti value was calculated by extrapolating the values to zero rate (Figure S1). Thermogravimetric analyses (TGA) were performed on a TA-Q50 (TA Instruments, New Castle, PA, USA), under air atmosphere with a heating rate of 20 °C/min from 30 °C to 800 °C.The morphology of the xLCN/SNP film was obtained by scanning electron microscopic (SEM) (FEI, Hillsboro, TX, USA).The tensile tests were measured by a dynamic mechanical analyzer (DMA) (TA Instruments, New Castle, PA, USA). For every sample, the force increased from 0.1 N to 18 N by a rate of 0.1 N/min at 30 °C, 90 °C, and 160 °C.

## 4. Conclusions

In this paper, a series of xLCNs/SiO_2_ composites with 1–7 wt% SiO_2_ nanoparitcles (SNP) were obtained. According to DSC, the Tg and Ti of the all xLCN/SNP samples were lower than that of pure LCN. Determined by TGA, the Td of the xLCN/SNP composites was always higher than that of pure LCN and exhibited a maximum increment of 35 °C by the addition of 1 wt% SiO_2_. Morphology investigation proved that the SiO_2_ nanoparticles were well dispersed. The stress-strain results showed that at a temperature above Tg or even above Ti, xLCN/SNP composites demonstrated higher tensile strength and the elongation at break. In summary, by incorporation the SiO_2_ nanoparticles into xLCN materials, xLCN/SNP composites with better thermal stability and mechanical property were achieved, which may improve the performance of the xLCNs during molding and reshaping process.

## Supplementary Materials

Supplementary File 1

## References

[1] Warner M., Terentjev E.M. (2003). Liquid Crystal Elastomers.

[2] Küpfer J., Finkelmann H. (1994). Liquid crystal elastomers: Influence of the orientational distribution of the crosslinks on the phase behaviour and reorientation processes. Macromol. Chem. Phys..

[3] Hogan P., Tajbakhsh A., Terentjev E. (2002). Uv manipulation of order and macroscopic shape in nematic elastomers. Phys. Rev. E.

[4] Ji Y., Marshall J.E., Terentjev E.M. (2012). Nanoparticle-liquid crystalline elastomer composites. Polymers.

[5] Marshall J.E., Gallagher S., Terentjev E.M., Smoukov S.K. (2013). Anisotropic colloidal micromuscles from liquid crystal elastomers. J. Am. Chem. Soc..

[6] Thomsen D.L., Keller P., Naciri J., Pink R., Jeon H., Shenoy D., Ratna B.R. (2001). Liquid crystal elastomers with mechanical properties of a muscle. Macromolecules.

[7] Tajbakhsh A., Terentjev E. (2001). Spontaneous thermal expansion of nematic elastomers. Eur. Phys. J. E.

[8] Yamada M., Kondo M., Mamiya J.-i., Yu Y., Kinoshita M., Barrett C.J., Ikeda T. (2008). Photomobile polymer materials: Towards light-driven plastic motors. Angew. Chem. Int. Ed..

[9] Amigó-Melchior A., Finkelmann H. (2002). A concept for bifocal contact—or intraocular lenses: Liquid single crystal hydrogels (“LSCH”). Polym. Adv. Technol..

[10] Sánchez-Ferrer A., Fischl T., Stubenrauch M., Wurmus H., Hoffmann M., Finkelmann H. (2009). Photo-crosslinked side-chain liquid-crystalline elastomers for microsystems. Macromol. Chem. Phys..

[11] Sánchez-Ferrer A., Fischl T., Stubenrauch M., Albrecht A., Wurmus H., Hoffmann M., Finkelmann H. (2011). Liquid-crystalline elastomer microvalve for microfluidics. Adv. Mater..

[12] Torras N., Zinoviev K.E., Esteve J., Sánchez-Ferrer A. (2013). Liquid-crystalline elastomer micropillar array for haptic actuation. J. Mater. Chem. C.

[13] Finkelmann H., Kim S.T., Palffy-Muhoray P., Taheri B. (2001). Tunable mirrorless lasing in cholesteric liquid crystalline elastomers. Adv. Mater..

[14] Pei Z., Yang Y., Chen Q., Terentjev E.M., Wei Y., Ji Y. (2014). Mouldable liquid-crystalline elastomer actuators with exchangeable covalent bonds. Nat. Mater..

[15] Ji Y., Huang Y.Y., Terentjev E.M. (2011). Dissolving and aligning carbon nanotubes in thermotropic liquid crystals. Langmuir.

[16] Ji Y., Huang Y.Y., Rungsawang R., Terentjev E.M. (2010). Dispersion and alignment of carbon nanotubes in liquid crystalline polymers and elastomers. Adv. Mater..

[17] Mukherjee P.K. (1997). Influence of non-mesogenic impurities on a nematic to isotropic phase transition. Liq. Cryst..

[18] Kaiser A., Winkler M., Krause S., Finkelmann H., Schmidt A.M. (2009). Magnetoactive liquid crystal elastomer nanocomposites. J. Mater. Chem..

[19] Haberl J.M., Sánchez-Ferrer A., Mihut A.M., Dietsch H., Hirt A.M., Mezzenga R. (2013). Liquid-crystalline elastomer-nanoparticle hybrids with reversible switch of magnetic memory. Adv. Mater..

[20] Domenici V., Zupančič B., Laguta V.V., Belous A.G., V’yunov O.I., Remškar M., Zalar B. (2010). PbTiO_3_ nanoparticles embedded in a liquid crystalline elastomer matrix: Structural and ordering properties. J. Phys. Chem. C.

[21] Domenici V., Conradi M., Remškar M., Viršek M., Zupančič B., Mrzel A., Chambers M., Zalar B. (2011). New composite films based on MoO_3-x_ nanowires aligned in a liquid single crystal elastomer matrix. J. Mater. Sci..

[22] Haberl J.M., Sánchez-Ferrer A., Mihut A.M., Dietsch H., Hirt A.M., Mezzenga R. (2013). Strain-induced macroscopic magnetic anisotropy from smectic liquid-crystalline elastomer-maghemite nanoparticle hybrid nanocomposites. Nanoscale.

[23] Ou C.-F., Shiu M.-C. (2010). Epoxy composites reinforced by different size silica nanoparticles. J. Appl. Polym. Sci..

[24] Alzina C., Sbirrazzuoli N., Mija A. (2011). Epoxy-amine based nanocomposites reinforced by silica nanoparticles. Relationships between morphologic aspects, cure kinetics, and thermal properties. J. Phys. Chem. C.

[25] Poulin P., Raghunathan V., Richetti P., Roux D. (1994). On the dispersion of latex particles in a nematic solution. I. Experimental evidence and a simple model. J. Phys. II.

[26] Anderson V., Terentjev E. (2001). Cellular solid behaviour of liquid crystal colloids 2. Mechanical properties. Eur. Phys. J. E.

[27] Halder S., Ghosh P.K., Goyat M.S., Ray S. (2013). Ultrasonic dual mode mixing and its effect on tensile properties of SiO_2_-epoxy nanocomposite. J. Adhes. Sci. Technol..

[28] Lai Y.-H., Kuo M., Huang J., Chen M. (2007). On the peek composites reinforced by surface-modified nano-silica. Mater. Sci. Eng.: A.

[29] Stöber W., Fink A., Bohn E. (1968). Controlled growth of monodisperse silica spheres in the micron size range. J. Colloid Interface Sci..

