# Supplementary material for: Liquid Crystalline Network Composites Reinforced by Silica Nanoparticles
Source: Materials (Basel). 2014 Jul 22;7(7):5356–65. doi: 10.3390/ma7075356 (PMC5455816; doi:10.3390/ma7075356)

Supplementary Information

Figure S1. DSC curves of samples with different SNP content (**a**) blank; (**b**) 1%; (**c**) 3%; (**d**) 5%; (**e**) 7%; (**f**) The linear fit of the Ti value.

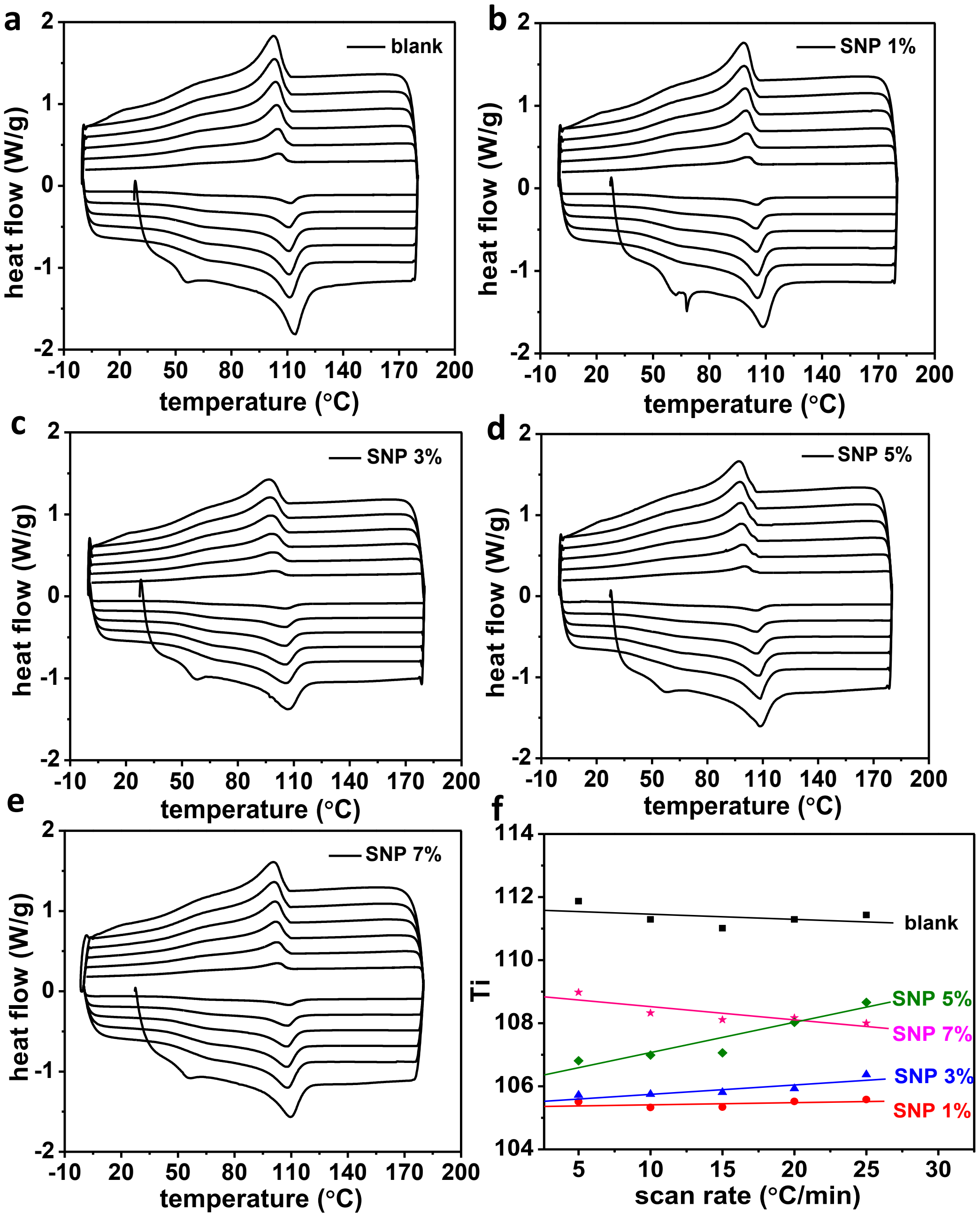

Figure S2. DLS distribution of SiO_2 nanoparticles in aqueous dispersion.

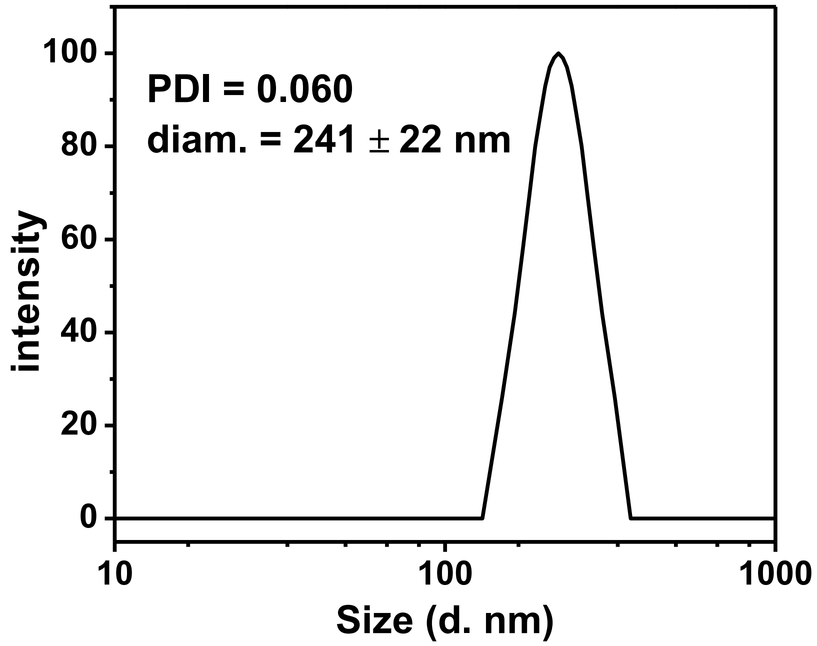

Figure S3. SEM picture of SiO_2 nanoparticles at a magnification of 30,000.

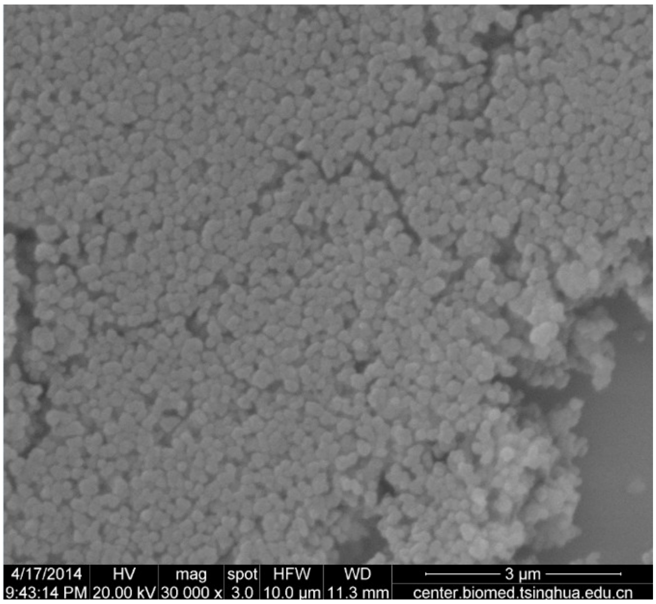

Supplement: Supplementary File 1 [file materials-07-05356-s001.pdf]
